# Impact of mRNA COVID-19 vaccination on hematological parameters in patients maintained on clozapine: A retrospective study from Qatar

**DOI:** 10.1016/j.rcsop.2025.100614

**Published:** 2025-05-18

**Authors:** Dalia Albahari, Oraib Abdallah, Shatha Mahmud Ismail Alqam, Mohammed Faisal Hamad Mohammed, Mohamed Ali Siddig Ahmed, Ovais Wadoo

**Affiliations:** a Department of Psychiatry, Hamad Medical Corporation, Qatar; b College of Medicine, Qatar University, Doha, Qatar

**Keywords:** Clozapine, Vaccination, COVID-19, Neutropenia, Leukopenia, Hematology, Safety

## Abstract

**Background:**

COVID-19 vaccines are known to cause transient changes in white blood cell counts as part of the immune activation process. Clozapine, an antipsychotic agent primarily prescribed for treatment-resistant schizophrenia, possesses both immunosuppressive and pro-inflammatory properties that may influence vaccine-related immune responses. The concurrent use of clozapine during COVID-19 vaccination has therefore raised concerns regarding potential hematological adverse effects. Despite increasing global research in this area, data from Arab populations remain scarce, highlighting the need for region-specific evidence. This study aimed to investigate the incidence of white blood cell and absolute neutrophil count abnormalities in patients receiving clozapine who were vaccinated with mRNA COVID-19 vaccines.

**Method:**

A retrospective study was conducted within Qatar's public mental health services. The study included patients on clozapine who received at least one dose of a COVID-19 vaccine between February 2020 and November 2022. Hematological parameters were assessed at three time points: pre-vaccination, shortly post-vaccination, and three months post-vaccination. Demographic, clinical, and vaccine-related factors were also examined.

**Results:**

Of 111 vaccinated patients, 74 had complete blood test data across the three time points. No cases of agranulocytosis or other serious hematological adverse effects were observed. Mild leukopenia occurred in 6.8–8.1 % of patients, and mild neutropenia in 5.4–6.8 %. These changes were transient and not associated with clozapine dosage, vaccine type, or other clinical variables.

**Conclusion:**

COVID-19 mRNA vaccines appear safe for individuals maintained on clozapine, with only minor, temporary changes in white blood cell counts. These findings support continued vaccination efforts in this population.

## Highlights

• Limited literature on COVID-19 vaccine safety in clozapine users in Arab countries is a critical research gap.
• Study confirms COVID-19 vaccines are safe for clozapine users, with only mild, temporary hematological changes.
• Research supports ongoing vaccination campaigns for individuals with severe mental illness.

## 1 Background

COVID-19 vaccines are known to induce transient changes in white blood cell (WBC) counts, such as mild lymphopenia, neutrophilia, and slight increase in monocytes, which reflect immune system activation.^1^ Following vaccination, lymphocytes may temporarily decline in peripheral blood due to their migration to lymphoid tissues. This move facilitates antigen recognition and the initiation of adaptive immune responses. Such transient lymphopenia indicates active immune engagement. Additionally, vaccination accelerates the release of cytokines like granulocyte colony-stimulating factor resulting in further neutrophil production and release from the bone marrow. This boosts the body's immediate defense mechanisms, representing a typical aspect of activation.^2^^,^^3^ However, clozapine, an antipsychotic medication, has potential immunosuppressive and pro-inflammatory effects, which may alter immune response.^4^ In individuals with severe mental illness (SMI), such as schizophrenia, there is an inherent immune dysregulation that can further influence the immune response to vaccinations.^5^ Evidence suggests that individuals with schizophrenia exhibit increased levels of pro-inflammatory cytokines (e.g., IL-6, TNF-α) and reduced anti-inflammatory cytokines. This imbalance could potentially lead to an exaggerated or insufficient immune response to vaccines.^5^ Moreover, they often experience chronic low-grade inflammation that may lead to reduced vaccine effectiveness, including lower antibody production and weaker cell-mediated immunity.^6^ Given that clozapine can exacerbate immune suppression, the combination of clozapine with a COVID-19 vaccine raises concerns regarding possible interactions that could lead to granulocytopenia, leukocytopenia, or lymphocytopenia. While international studies have generally reported mild and temporary changes in WBC counts following vaccination in patients on clozapine, ^7^ the specific effects in the Arab population have not been studied. The absence of regional studies on the impact of COVID-19 vaccines in patients maintained on clozapine highlights critical gap in research. Given the unique genetic, environmental, and healthcare system factors in Arab populations, understanding how these variables affect vaccine outcomes is essential for optimizing vaccination policy and strategy. By addressing this gap, this research can help inform healthcare policies, ensuring that patients maintained on clozapine receive appropriate guidance for vaccination, and contributing to broader efforts to optimize vaccine safety and efficacy in vulnerable populations such as those on clozapine with comorbidities across the Arab world. This study was aimed at investigating the incidence of white blood cell and absolute neutrophilic count abnormalities in mRNA COVID-19- vaccinated patients on clozapine.

## 2 Materials and methods

### 2.1 Ethics statement

The study (protocol number MRC 01–22-768) was approved by the local institutional review board.

### 2.2 Study design

This is a retrospective cohort study.

### 2.3 Setting

The Mental Health Services (MHS) of Hamad Medical Corporation is Qatar's major provider of public mental health services. MHS is the only licensed health center in the country that can prescribe clozapine.^8^^,^^9^ MHS's pharmacy department maintains a registry of all clozapine patients and ensures that laboratory monitoring and medication criteria are followed.

### 2.4 Sample

The study included all patients from the Mental Health Services of Hamad Medical Corporation in Qatar who were on clozapine and received at least one dose of a COVID-19 vaccine between February 29, 2020, and November 28, 2022. No sample size was calculated as it was a cohort sampling and there were no exclusions. In terms of vaccines, Qatar's Ministry of Public Health approved four COVID-19 vaccines (Pfizer/BioNTech, Moderna, AstraZeneca, and Janssen -Johnson & Johnson).

### 2.5 Data collection

Medical records of all patients who dispensed clozapine between February 29, 2020, and November 28, 2022, were retrieved from the pharmacy department of Mental Health Services. The research team analyzed electronic health records (Cerner®)and looked into documentation notes, laboratory views and medication charts for the following variables and outcomes:

1. Demographic Data, including age, gender, nationality, BMI and smoking status.
2. COVID-19 Vaccination Status: Documentation of vaccination status and the specific type of vaccine administered.
3. Automated White Blood Cell (WBC) Count & Absolute Neutrophil Count (ANC): WBC & ANC results were examined at three intervals for patients meeting the inclusion criteria: 7–30 days prior to vaccination; first available results after vaccination; three months following the final vaccine dose. [Leukopenia was defined as WBC <4 × 10^9^/L. Neutropenia was classified as follows: Mild neutropenia: ANC between 1.5 and 1 × 10^9^/L, Moderate neutropenia: ANC between 0.9 and 0.5 × 10^9^/L, Severe neutropenia: ANC < 0.5 × 10^9^/L, Agranulocytosis: ANC < 0.1 × 10^9^/L] in accordance with local laboratory standards. Ther recommended monitoring frequency and clinical decisions by ANC level for the general patient population is based on the prescribing information by FDA (Fig. 1).^10^

**Fig. 1:**
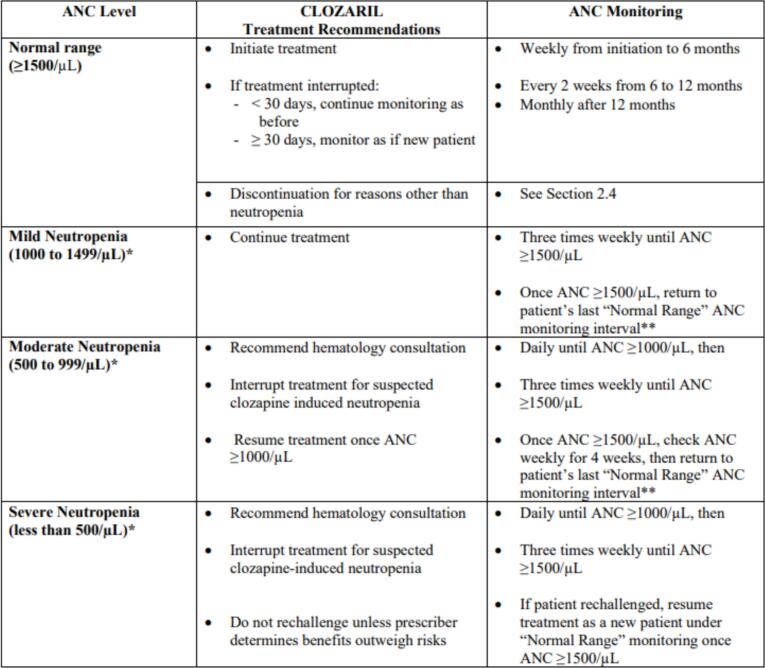
Clozapine Treatment recommendations based on ANC monitoring for general population.^10^
4. Benign Ethnic Neutropenia (BEN): Diagnosed in patients with persistently low ANC (<1.5 × 10^9^/L) without evidence of infection or adverse effects. This condition, common in certain ethnic groups from the Middle East and among individuals of African descent, is a key consideration when interpreting ANC levels during clozapine treatment.
5. Clozapine Dose: The dose at the time of vaccination was recorded.
6. Chronic Illness Diagnoses: Documentation of comorbid chronic conditions, including chronic cardiovascular disease, chronic respiratory disease, diabetes mellitus.
7. Psychiatric diagnosis as per Diagnostic and Statistical Manual of Mental Disorders, Fifth Edition.^11^
8. Other concurrent antipsychotics medications given with clozapine (if any).

### 2.6 Data analysis

Data was analyzed using SPSS version 29. Descriptive statistics were applied (mean, standard deviation, median, and range). Categorical variables were described as frequency and percentages. Data were explored for outliers, skewness, and normality. Shapiro-Wilk test for normality was performed. Data was not normally distributed, so non-parametric tests were used. The Wilcoxon signed-rank test was performed to assess whether there is a statistically significant difference between the paired observations (WBC and ANC before and after the first and second doses of the COVID-19 vaccine). Tests were considered statistically significant when a two-sided *p*-value of less than 0.05. Moreover, the demographics for those with neutropenia were compared to those without neutropenia upon receiving the COVID-19 vaccine using Fisher's Exact Test (for categorical variables) and Mann-Whitney *U* Test (continuous variables). All statistical comparisons were performed using available data without imputation, as the proportion of missing data was minimal and unlikely to significantly impact the results.

## 3 Results

During the study period, 111 out of 157 patients of those who are prescribed clozapine (70.7 %) received two doses of a COVID-19 vaccine, with the majority (77 %) receiving the BNT162b2 vaccine. A total of 74 vaccinated patients (66.6 %) had complete hematological data and were included in the analysis. Patient demographics and clinical characteristics are detailed in Table 1. The median age for those who received the vaccine was 38 with IQR of 30–44.5. The majority were adults between 30 and 49 years. The median body mass index was 31 with IQR of 26.03–35.63. Males were predominant (*n* = 51, 68.92 %) and 59.46 % where Qatari. In total, 35.14 % of them were active smokers. Forty-four (59.46 %) were on clozapine monotherapy. Additionally, median and IQR of clozapine dose upon 1st and 2nd vaccine dose in mg/d was: 337.5(200–450) and 350 (200–481.45), respectively.

**Table 1:** Demographics and clinical characteristics of COVID-19 vaccinated patients with neutropenia vs without neutropenia post vaccines.

Parameters	n (%) for those who received COVID-19 vaccine (*n* = 74)	With neutropenia (n = 7)	Without neutropenia (*n* = 67)	P- value
**Age**^&^ >60 20–29 30–39 40–49 50–59	2(2.70 %) 16(21.62 %) 24(32.43 %) 24(32.43 %) 8(10.81 %)	0 4 1 1 1	2 12 23 23 7	0.173⁎⁎
**Gender**				0.375⁎
Female	23(31.08 %)	3	20	
Male	51(68.92 %)	4	47	
**Nationality**				0.759⁎⁎
Qatari	44 (59.46 %)	2	42	
Non-Qatari	30 (40.54 %)	1	29	
**Smoking Status** Current smoker Ex-smoker Never smoked unable to identify	26 (35.14 %) 1(1.35 %) 41(55.41 %) 6 (8.11 %)	1 0 6 0	25 1 35 6	0.396⁎⁎
**Schizophrenia diagnosis**	67(90.54 %)	7	60	0.668⁎⁎
**Chronic respiratory disease diagnosis**	7 (9.46 %)	1	6	0.517⁎
**Chronic Cardiovascular disease diagnosis**	2(2.70 %)	0	2	0.819⁎
**Diabetes Mellitus**	9 (12.16 %)	2	7	0.200⁎
**BNT162b2 vaccine**	57(77.03 %)	5	52	0.510⁎
**Medications upon vaccination**				
Clozapine alone	44(59.46 %)	3	41	0.292⁎
Clozapine with other antipsychotics	30(40.54 %)	4	26	

*Fisher's exact test; ** Pearson Chi-square, ^&^ Age Median (IQR): 38(30–44.25).

Furthermore, there were no statistically significant demographic or clinical characteristics were found between the patients who experienced neutropenia and those who did not (Table 1).

### 3.1 WBC and ANC levels before and after COVID-19 vaccination

The laboratory results of each patient were checked at three time points (7–30 days pre-vaccination, the next available WBC results post-vaccination, and three months after the last vaccine dose). None of these patients had a benign ethnic neutropenia diagnosis. None of the patients experienced agranulocytosis. Nine incidents of neutropenia were identified following vaccination in seven patients. Three patients experienced neutropenia after the two vaccine doses. Five patients experienced mild leukopenia (6.8 %) upon receiving the first vaccine, and four experienced mild neutropenia (5.4 %). Six patients experienced mild leukopenia (8.1 %) on the second vaccine dose, and five experienced mild neutropenia (6.8 %). All WBC and ANC values were normal within three months after the last vaccine dose. Changes in WBC and ANC levels before and after receiving COVID-19 vaccinations were insignificant (Table 2).

**Table 2:** WBC and ANC levels before and after the 1st and 2nd COVID-19 vaccine doses.

	Pre- COVID-19 vaccine	Post-COVID-19 vaccine	P-value ^&^
**WBC (median IQR) -1st dose**	7.2 × 10^9^/L (5.6–8.7) *	6.8 × 10^9^/L (5.65–8.45) **	0.771
**ANC (median IQR)- 1st dose**	4 × 10^9^/L (2.8–5.4) *	3.4 × 10^9^/L (2.8–5.2) **	0.417
**WBC (median IQR)- 2nd dose**	6.75 × 10^9^/L (5.5–8.5) ^^^	6.9 × 10^9^/L(5.45–8.25) ^$^	0.082
**ANC (median IQR)- 2nd dose**	3.5 × 10^9^/L (2.85–5.4) ^Ω^	3.4 × 10^9^/L (2.7–4.9) ^$^	0.079

*(n) = 63, **(n) = 65 & Wilcoxon Signed Ranks Test, Significance level 0.05.

^(n) = 58, ^$^(n) = 61, Ω (n) = 57 & Wilcoxon Signed Ranks Test, Significance level 0.05.

## 4 Discussion

Globally, COVID-19 immunization has had little hematological effects in patients receiving clozapine, with only temporary changes in WBC and ANC levels. Veerman et al. discovered mild granulocytopenia (3 % and 5 %), moderate granulocytopenia (1 % and 0 %), and leukocytopenia (2 % and 3 %) in 139 clozapine patients after receiving COVID-19 vaccinations, with no clinical effects.^12^ Lim et al. reported one (0.8 %) and two (1.7 %) cases of asymptomatic moderate neutropenia following the first and second doses, respectively, with spontaneous recovery.^13^ These three patients had a history of granulocytopenia. A recently published systematic review and analysis of four databases supplemented by adverse events analysis of 137 patients found no serious safety concerns specific to COVID-19 vaccines with clozapine.^14^ All the studies exploring impact of COVID-19 vaccines in patients maintained on clozapine in non-Arab populations, report minimal effects on white blood cell counts.^15^, ^16^, ^17^, 18., 19, 20, 21

This is the first study of its kind in the Arab population, and it is critical because genetic, environmental, and healthcare system factors can all influence immune responses and vaccination results. For example, variations in genes encoding cytokines, can alter immune activation and regulation.^22^ This can directly impact vaccine efficacy and susceptibility to adverse reactions. Additionally, immunogenicity of vaccine and its adverse events have been stated at higher rates for females than males for multiple vaccines.^23^ More importantly, taking into the regional prevalence for BEN, it is widely spread in Middle Eastern and African populations and should be considered when interpreting ANC values in clozapine-treated patients.^24^^,^^25^ Furthermore, genetic polymorphisms impacting drug metabolism, such as variations in the CYP450 enzyme system, can produce differences in clozapine processing and immunological effects.^26^^,^^27^ Environmental variables, such as insufficient sun exposure, which contributes to vitamin D deficiency, can also influence immune responses. Vitamin D plays a crucial role in modulating both innate and adaptive immune responses, and its deficiency is associated with increased susceptibility to infections.^28^ Obesity also influences immune function, potentially altering immune responses.^29^

The study found no significant differences in hematological outcomes and the conclusions are consistent with international data. The controlled monitoring of WBC and ANC at predetermined intervals reinforces the argument that any observed alterations were transient and unrelated to vaccine schedule. By focusing on a single-provider healthcare system, the study reduces variability in treatment and monitoring, resulting in a more reliable assessment than multicenter international studies. Furthermore, consistent with earlier studies, this study found no significant differences in hematological outcomes based on vaccine type, specifically mRNA vaccines (BNT162b2 and mRNA-1273), or demographic and clinical risk factors such as age, gender, comorbidities, and clozapine dose. This study's population-specific findings, particularly the lack of BEN, are surprising. Overall, the results of this study support the continued use of mRNA COVID-19 vaccines in Arab patients.

Our sample primarily consisted of men, which may not fully represent the typical gender distribution of mental disorders. While global data indicate a higher prevalence of mental disorders in women, cultural factors and healthcare access patterns in Arab contexts may influence gender representation in clinical samples. Further research is needed to explore gender-specific differences and the factors shaping mental healthcare utilization in Arab populations.

Interestingly, as the administration of the vaccine was not associated with significant changes. Another study found no substantial difference in the rate of severe infection, hospitalization rates and length of inpatient stay for patients on clozapine versus those with non-clozapine. These outcomes suggest a recurring tendency across different aspects of the vaccine's effect.^30^

### 4.1 Limitations

The results may be limited by the incomplete laboratory test profiles of clozapine patients, which reduced the study population. The lack of laboratory tests could be related to the extended hematological monitoring frequency and the mobility restrictions imposed during parts of the COVID-19 pandemic.^31, 32^ The study results are limited to two vaccines (mRNA-1273, BNT162b2) and may not be generalizable to other types of vaccines. As a retrospective cohort study, this design inherently limits causal inferences, as it primarily identifies associations rather than establishing direct cause-and-effect relationships. Future research should prioritize prospective longitudinal cohort studies to assess the long-term effects of COVID-19 vaccination on blood parameters. Additionally, comparative studies examining different antipsychotic medications would enhance the generalizability of the findings.

## 5 Conclusion

Changes in WBC counts following COVID-19 vaccination in patients on clozapine were not clinically significant, did not necessitate additional hematological monitoring, and did not pose a barrier to vaccination. Prioritizing vaccination in patients maintained on clozapine is likely protective; however, clinicians should remain vigilant for potential adverse drug events. This study contributes to the limited body of research on neutropenia following COVID-19 vaccination in clozapine-treated patients. The findings support clinical decision-making and add to the growing evidence base that may inform future clinical guidelines.

## CRediT authorship contribution statement

**Dalia Albahari:** Writing – review & editing, Writing – original draft, Visualization, Validation, Supervision, Resources, Project administration, Methodology, Investigation, Formal analysis, Data curation, Conceptualization. **Oraib Abdallah:** Writing – review & editing, Visualization, Validation, Software, Methodology, Investigation, Formal analysis, Data curation. **Shatha Mahmud Ismail Alqam:** Writing – review & editing, Visualization, Validation, Data curation. **Mohammed Faisal Hamad Mohammed:** Writing – review & editing, Visualization, Data curation. **Mohamed Ali Siddig Ahmed:** Writing – review & editing, Visualization, Validation, Resources, Conceptualization. **Ovais Wadoo:** Writing – review & editing, Writing – original draft, Visualization, Validation, Supervision, Resources, Project administration, Methodology, Investigation, Conceptualization.

## Funding

This research did not receive any specific grant from funding agencies in the public, commercial, or not-for-profit sectors. The open access publication fees was paid by the Medical Research Centre, Hamad Medical Corporation, Qatar.

## Declaration of competing interest

OW has received honorarium from Janssen,Viatris,Newbridge which is not related to the content of this research or publication. All other authors have no conflict of interest.
